# Nanomechanical Sensors for Gas Detection towards Artificial Olfaction

**DOI:** 10.3390/bios12040256

**Published:** 2022-04-18

**Authors:** Kosuke Minami

**Affiliations:** Olfactory Sensors Group, Center for Functional Sensor & Actuator (CFSN), Research Center for Functional Materials, National Institute for Materials Science (NIMS), 1-1 Namiki, Tsukuba 305-0044, Japan; minami.kosuke@nims.go.jp

Humans, as well as other organisms, tend to recognize their surroundings by smells/odors. Each smell/odor is usually composed of dozens to thousands of different types of molecules out of more than 400,000 types of odorous/odorless molecules [1]. We detect such a complex smell/odor as a simultaneous interaction with our olfactory receptors and recognize the small/odor by comprehensively analyzing the signals mediated by various receptors in the brain. Persaud et al. proposed a concept of artificial olfaction using an array of different types of sensors and resultant unique signal patterns to discriminate the specific smells/odors [2,3]. This basic concept of artificial olfaction can be divided into some components corresponding to our olfactory system: receptor materials as olfactory receptors, sensing elements and transducers as olfactory cells, and pattern recognition analysis as neural activity of the brain. Accordingly, to realize artificial olfaction, gas sensors are the key components in addition to data processing technologies, including artificial intelligence and machine learning algorithms [4,5]. As we have over 400 different species of olfactory receptors in our noses [6], the development of artificial olfaction requires a variety of receptor materials with wide selectivity and sensitivity.

Over the last few decades, nanomechanical sensors have received significant attention as a powerful tool for gas detection and hence for artificial olfaction. Their potential applications range from food, environmental, and healthcare monitoring to medical diagnostics [7,8,9]. Nanomechanical sensors, including both dynamic and static mode operations, detect volume- and/or mass-induced mechanical changes in a sensing element [9]. Since it has been observed that almost all solid materials exhibit mechanical deformation upon gas sorption, various types of solid materials can be utilized as receptor materials, providing a wide range of chemical selectivity and sensitivity. To realize artificial olfaction, it is required to integrate all related technologies in multiple fields into one system [3], including the fabrication of versatile receptor materials, the construction of highly sensitive nanomechanical sensor systems, and the development of effective data processing algorithms.

Therefore, not limited to the research on artificial olfaction, all related research articles and comprehensive reviews are welcome to the current Special Issue entitled “Nanomechanical Sensors for Gas Detection”. This Special Issue aims to integrate all knowledge in the wide-ranging fields to realize practical artificial olfaction.
